# A unique peptide-based pharmacophore identifies an inhibitory compound against the A-subunit of Shiga toxin

**DOI:** 10.1038/s41598-022-15316-1

**Published:** 2022-07-06

**Authors:** Miho Watanabe-Takahashi, Miki Senda, Ryunosuke Yoshino, Masahiro Hibino, Shinichiro Hama, Tohru Terada, Kentaro Shimizu, Toshiya Senda, Kiyotaka Nishikawa

**Affiliations:** 1 Department of Molecular Life Sciences, Graduate School of Life and Medical Sciences, Doshisha University, Kyoto, Japan; 2 Structural Biology Research Center, Institute of Materials Structure Science, High Energy Accelerator Research Organization (KEK), Ibaraki, Japan; 3 Department of Biotechnology, Graduate School of Agricultural and Life Sciences, The University of Tokyo, Tokyo, Japan; 4 Transborder Medical Research Center, University of Tsukuba, Ibaraki, Japan; 5 Department of Materials Structure Science, School of High Energy Accelerator Science, The Graduate University of Advanced Studies (Soken-dai), Ibaraki, Japan

**Keywords:** Bacterial toxins, Molecular modelling, X-ray crystallography

## Abstract

Shiga toxin (Stx), a major virulence factor of enterohemorrhagic *Escherichia coli* (EHEC), can cause fatal systemic complications. Recently, we identified a potent inhibitory peptide that binds to the catalytic A-subunit of Stx. Here, using biochemical structural analysis and X-ray crystallography, we determined a minimal essential peptide motif that occupies the catalytic cavity and is required for binding to the A-subunit of Stx2a, a highly virulent Stx subtype. Molecular dynamics simulations also identified the same motif and allowed determination of a unique pharmacophore for A-subunit binding. Notably, a series of synthetic peptides containing the motif efficiently inhibit Stx2a. In addition, pharmacophore screening and subsequent docking simulations ultimately identified nine Stx2a-interacting molecules out of a chemical compound database consisting of over 7,400,000 molecules. Critically, one of these molecules markedly inhibits Stx2a both in vitro and in vivo, clearly demonstrating the significance of the pharmacophore for identifying therapeutic agents against EHEC infection.

## Introduction

Infection with enterohemorrhagic *Escherichia coli* (EHEC), which includes the O157:H7 serotype, causes gastrointestinal symptoms, such as bloody diarrhea and hemorrhagic colitis^1–3^. Critically, these are often complicated by fatal systemic sequelae, including acute encephalopathy and hemolytic uremic syndrome, the leading cause of acute renal failure in children^4,5^. EHEC strains produce Shiga toxin (Stx), which induces damage to the intestinal lining and acts as a key mediator of bacterial pathogenesis^6,7^. Thus, effective inhibitors of Stx represent a promising class of therapeutic agents against EHEC infection.

Stx molecules are classified into two closely related subgroups, known as Stx1 and Stx2, each of which further contain various subtypes, including the two major subtypes, Stx1a and Stx2a^8–10^. Of these, Stx2a, is more virulent and has been linked to fatal systemic complications in humans^11^. All Stx proteins consist of a catalytic A-subunit and a B-subunit pentamer. The catalytic A-subunit, which is a member of ribosome-inactivating proteins (RIPs), has an RNA *N*-glycosidase activity that cleaves a specific adenine from 28S ribosomal RNA to inhibit eukaryotic protein synthesis^12^. The B-subunit pentamer functions to bind Galα[1–4]–Galβ[1–4]–Glcβ-ceramide (Gb3), a glycolipid present on the surface of target cells^7,13^. Each B-subunit has three distinctive binding sites (i.e., sites 1, 2, and 3) for the trisaccharide moiety of Gb3^14,15^. Critically, this contributes to formation of a multivalent interaction that markedly increases binding affinity by a million-fold—a phenomenon referred to as the “clustering effect.”

Previously, we developed a library of tetravalent peptides designed to exhibit the clustering effect^16^. Affinity-based screening of this library identified a series of tetravalent peptides that bind to the B-subunit pentamer with high affinity and inhibit Stx toxicity in vitro and in vivo^16,17^. One of these tetravalent peptides, MMβA-tet, which has the synthetic amino acid βAla in its motif, inhibits both Stx1a and Stx2a with the greatest potency^18^. Unexpectedly, we further found that a monomeric peptide with the same motif as MMβA-tet (referred to as MMβA-mono), also inhibits cytotoxicity of Stx1a and Stx2a, although it cannot exert the clustering effect nor bind to the Stx B-subunit pentamer. Crystallographic analysis revealed that MMβA-mono binds to the A-subunit of Stx2a and fully occupies its catalytic cavity. Notably, MMβA-mono occupies a wider region of the catalytic cavity, interacting with residues Val78, Asp94, Ser112, Tyr114, Thr115, Glu167, and Arg170, relative to previously developed small molecule inhibitors that interact only with Val78, Ser112, and Arg170^19^ in the “adenine-specificity” pocket of the A-subunit^20^. Asp94 and Glu167 in particular, which are located in the gate area and in the bottom of the catalytic pocket, respectively, have never been demonstrated as drug targets. Thus, we have shown that the MMβA peptide motif demonstrates potent inhibition of two functionally distinct subunits of Stx, the A- and the B-subunits, depending on the organization of the peptide structure.

In this study, we identified a minimal essential motif of MMβA-mono required for binding to and inhibition of the A-subunit of Stx2a. In addition, using both experimental analysis and also molecular dynamics (MD) simulations, a computational method to predict physical movements of biomolecules based on the Newtonian equation of motion^21,22^, we determined a unique pharmacophore for binding to key residues of Stx2a. Based on this pharmacophore, virtual screening of chemical database of small compounds successfully identified a molecule that efficiently inhibits toxicity of Stx2a in vitro and in vivo. Thus, we predict that this molecule hold potential as a new, promising therapeutic agent for EHEC infection.

## Results

### Shorter peptides derived from MMβA-mono effectively bind to the A-subunit of Stx2a

**Figure 1:**
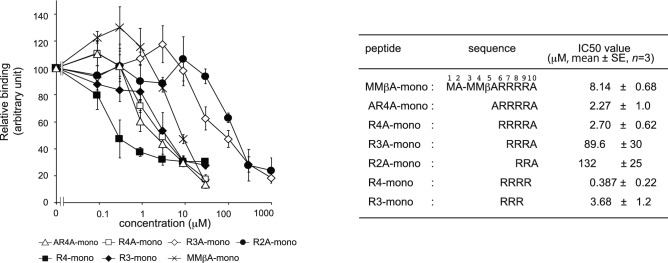
A series of shorter MMβA-mono-derived peptides inhibit binding between the Stx2a A-subunit and MMβA-mono. The inhibitory effects of a series of shorter MMβA-mono-derived peptides on binding between the Stx2a A-subunit and MMβA-mono were measured using the AlphaScreen assay. Relative binding is shown in the left panel, and peptide amino acid sequences and IC50 values are shown in the right panel. Data are presented as a percentage of the control value without peptides (mean ± standard error [SE], *n* = 3).

MMβA-mono is a 10-residue peptide with the sequence, Met-Ala-Met-Met-βAla-Arg-Arg-Arg-Arg-Ala, in which βAla is synthetic amino acid. This peptide has been shown to bind exclusively to the Stx2a A-subunit (apparent Kd = 0.05 µM), but not to the B-subunit pentamer^18^. To determine a minimal essential motif of MMβA-mono that binds to the Stx2a A-subunit, we prepared a series of shorter peptides based on the sequence of MMβA-mono and measured their ability to inhibit binding of MMβA-mono to the A-subunit using the AlphaScreen assay. We found that R4-mono, AR4A-mono, R4A-mono, and R3-mono exhibit greater inhibitory effects (relative IC50 values = 0.387, 2.27, 2.70, and 3.68 µM, respectively) than non-tagged MMβA-mono (relative IC50 = 8.14 µM), which was used as a positive control to compete the binding (Fig. 1). Notably, R3A-mono and R2A-mono (relative IC50 values = 89.6 and 132 µM, respectively) also substantially inhibit binding, albeit with efficacies less than that of MMβA-mono (Fig. 1).

**Figure 2:**
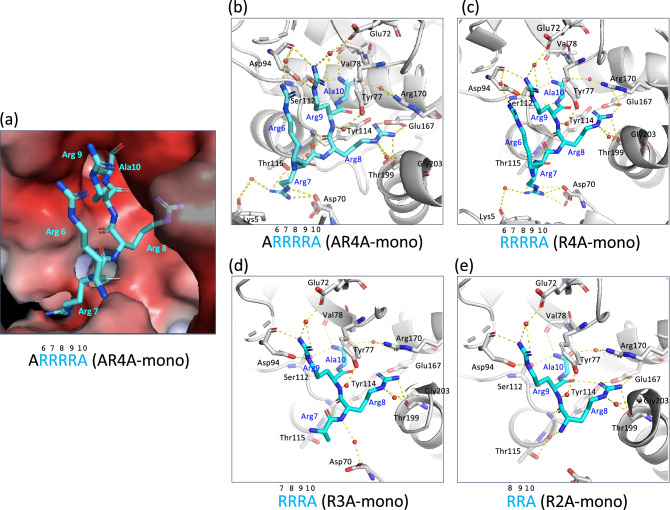
Structural analysis of the interaction between shorter MMβA-mono-derived peptides and the Stx2a A-subunit. (**a**) Close-up view of the Stx2a holotoxin in complex with AR4A-mono. Stx2a is shown as a charge distribution surface model, with the surface colored by charge (blue, positive; red, negative). AR4A-mono is shown as a stick model. (**b–e**) Structural view of binding between the A-subunit and shorter MMβA-mono-derived peptides (**b**, AR4A-mono; **c**, R4A-mono; **d**, R3A-mono; and **e,** R2A-mono). In R3A-mono case, the electron density for the side chain of Arg7 was not determined (see also Supplementary Fig. S1). Interacting residues are shown as stick models, hydrogen bonds are shown as broken lines, and water molecules are shown as spheres. All crystal structure images were created using PyMOL ver. 2.3.4, (https://pymol.org/2/).

To elucidate the precise manner by which these shorter peptides bind to Stx2a, X-ray crystallography analysis was performed with co-crystals of each peptide and the Stx2a holotoxin, which were obtained via the soaking method. Data collection and refinement statistics are summarized in Table 1. We were able to determine electron densities for AR4A-mono, R4A-mono, R3A-mono, and R2A-mono (Supplementary Fig. S1), and X-ray crystal structures were solved to final resolutions of 1.80, 1.80, 1.90, and 1.75 Å-resolution, respectively. These structures reveal that the AR4A-mono, R4A-mono, R3A-mono, and R2A-mono peptides bind tightly to the catalytic A-subunit (Fig. 2). In all cases, the common Arg8-Arg9-Ala10 motif (numbering reflects residue position in full-length MMβA-mono) interacts equally with the Glu72, Tyr77, Val78, Asp94, Ser112, Tyr114, Thr115, Glu167, Arg170, Thr199, and Gly203 residues present in the catalytic pocket of the A-subunit, whereas the Arg8 and Arg9 residues of the motif electrostatically interact with Glu167 and Asp94, respectively (Fig. 2, Table 2). The amide group of the Ala10, but not its side chain, interacts with the Val78, Ser112 and Arg170 residues. In addition, Arg6 and Arg7 of both AR4A-mono and R4A-mono electrostatically interact, respectively, with Asp94 of the A-subunit and Asp70 of the B-subunit, which is adjacent to the catalytic cavity. The main chain of Arg8 of R3A-mono also electrostatically interacts with Asp70 of the B-subunit. Overall, we find that the binding patterns of these shorter peptides are almost identical to that of MMβA-mono^18^. Combined with results from the competition assay, these data demonstrate that these shorter peptides efficiently bind to the A-subunit of Stx2a, and R2A-mono is the minimal essential motif needed for binding.

**Table 1:** Data collection and refinement statistics.

	AR4A-mono	R4A-mono	R3A-mono	R2A-mono
**Data collection**				
Space group	*P*6_1_	*P*6_1_	*P*6_1_	*P*6_1_
Cell dimensions				
*a*, *b*, *c* (Å)	146.2, 146.2, 60.2	146.4, 146.4, 60.7	146.1, 146.1, 60.8	146.2, 146.2, 60.5
*α*, *β*, *γ* (°)	90, 90, 120	90, 90, 120	90, 90, 120	90, 90, 120
Resolution (Å)	47.86–1.80 (1.84–1.80)	47.92–1.80 (1.84–1.80)	47.82–1.90 (1.94–1.90)	47.85–1.75 (1.78–1.75)
R_pim_	0.039 (0.250)	0.035 (0.268)	0.062 (0.273)	0.035 (0.247)
I/σ (I)	17.7 (3.1)	18.2 (3.0)	10.7 (3.0)	20.9 (3.4)
Completeness (%)	100.0 (100.0)	100.0 (100.0)	94.7 (100.0)	100.0 (100.0)
Redundancy	21.2 (21.3)	21.2 (21.4)	18.5 (21.0)	20.9 (3.4)
**Refinement**				
Resolution (Å)	47.86–1.80	47.92–1.80	46.73–1.90	47.85–1.75
No. reflections	68,212	68,893	55,253	74,504
R_free_ / R_work_	0.195/0.166	0.191/0.167	0.237/0.193	0.186/0.166
No. atoms				
Protein	4936	4947	4905	4928
Peptide	50	50	33	28
PPS	52	52	52	52
Water	492	490	396	429
B-factors				
Protein (Å^2^)	15.9	17.7	20.5	15.0
Peptide (Å^2^)	25.5	25.5	18.4	13.6
PPS (Å)	34.9	28.7	30.8	22.7
Water (Å)	24.5	26.2	26.4	22.3
r.m.s deviations				
Bond lengths (Å)	0.007	0.007	0.007	0.006
Bond angles (°)	0.876	0.863	0.935	0.848
Ramachandran plot				
Favored/Allowed/Outliers	99.0/1.0/0.0	98.9/1.1/0.0	98.6/1.5/0.0	99.0/1.0/0.0
PDB code	7VHC	7VHD	7VHE	7VHF

Values in parentheses are for highest-resolution shell. Each dataset was collected from one crystal.

**Table 2:** Hydrogen bonding interactions between shorter peptides and the Stx2a A-subunit.

Peptide	Wat	Stx2a	Hydrogen bond		Distance			
			Donor	Acceptor	AR4A	R4A	R3A	R2A
Arg6	Wat	Asp94	Arg6 NH2	Wat O	2.61	2.78	–	–
Arg6	Wat	Asp94	Wat101	Asp94 O	2.85	2.82	–	–
Arg7		Thr115	Arg7 NH2	Thr115 OG1	3.26	3.30	–	–
Arg7		Thr115	Arg7 NH1	Thr115 OG1	3.25	3.30	–	–
Arg7		Asp70*	Arg7 NH2	Asp70 OXT	3.21	3.19	–	–
Arg7		Asp70*	Arg7 NE	Asp70 OXT	3.14	3.43	–	–
Arg7	Wat	Lys5*	Arg7 NH1	Wat102 O	2.88	2.87	–	–
Arg7	Wat	Lys5*	Wat102	Lys5 O	3.36	3.65	–	–
Arg8		Tyr114	Tyr114 N	Arg8 O	3.00	2.96	3.07	2.83
Arg8	Wat	Thr115	Arg8 N	Wat104	3.42	3.49	3.34	2.95
Arg8	Wat	Thr115	Wat104	Thr115 OG1	2.90	2.89	2.99	2.99
Arg8	Wat	Asp70*	Arg8 N	Wat305	–	–	3.00	–
Arg8	Wat	Asp70*	Wat305	Asp70 O	–	–	2.51	–
Arg8		Thr199	Arg8 NH2	Thr199 O	2.93	2.92	2.96	2.96
Arg8		Thr199	Arg8 NE	Thr199 O	3.10	3.09	3.12	3.11
Arg8		Glu167	Arg8 NH2	Glu167 OE1	3.04	2.98	3.02	3.03
Arg8		Glu167	Arg8 NH1	Glu167 OE2	2.92	2.84	2.88	2.92
Arg8	Wat	Gly203	Gly203 N	Wat105 O	3.04	3.05	3.09	3.05
Arg8	Wat	Gly203	Arg8 NE	Wat105 O	2.99	3.12	3.06	3.00
Arg9		Asp94	Arg9 NE	Asp94 OD2	2.95	2.99	2.91	2.94
Arg9		Asp94	Arg9 NH2	Asp94 O	2.99	2.94	2.86	2.94
Arg9	Wat	Tyr77	Arg9 N	Wat318 O	2.92	2.88	2.71	2.87
Arg9	Wat	Tyr77	Wat318 O	Tyr77 O	2.60	2.54	2.60	2.89
Arg9	Wat	Glu72	Arg9 NH2	Wat346	2.92	2.78	2.77	2.88
Arg9	Wat	Glu72	Arg9 NH1	Wat346	3.14	3.07	2.89	3.02
Arg9	Wat	Glu72	Wat346 O	Glu72 OE2	2.70	2.63	2.77	2.70
Ala10		Val78	Val78 N	Ala10 O	2.75	2.79	2.77	2.80
Ala10		Ser112	Ala10 NT	Ser112 OG	2.97	2.98	2.97	2.93
Ala10		Val78	Ala10 NT	Val78 O	2.96	2.95	2.93	2.83
Ala10		Ser112	Ala10 N	Ser112 O	2.83	2.81	2.85	2.83
Ala10	Wat	Arg170	Arg170 NH1	Wat103 O	2.85	2.80	2.88	2.86
Ala10	Wat	Arg170	Arg170 NH2	Wat103 O	3.07	3.04	3.01	2.99
Ala10	Wat	Arg170	Wat103 O	Ala10 O	2.70	2.86	2.85	2.77

*Asterisk indicates the residue is in the Stx2a B-subunit.

### Determination of a pharmacophore to bind to the A-subunit of Stx2a

**Figure 3:**
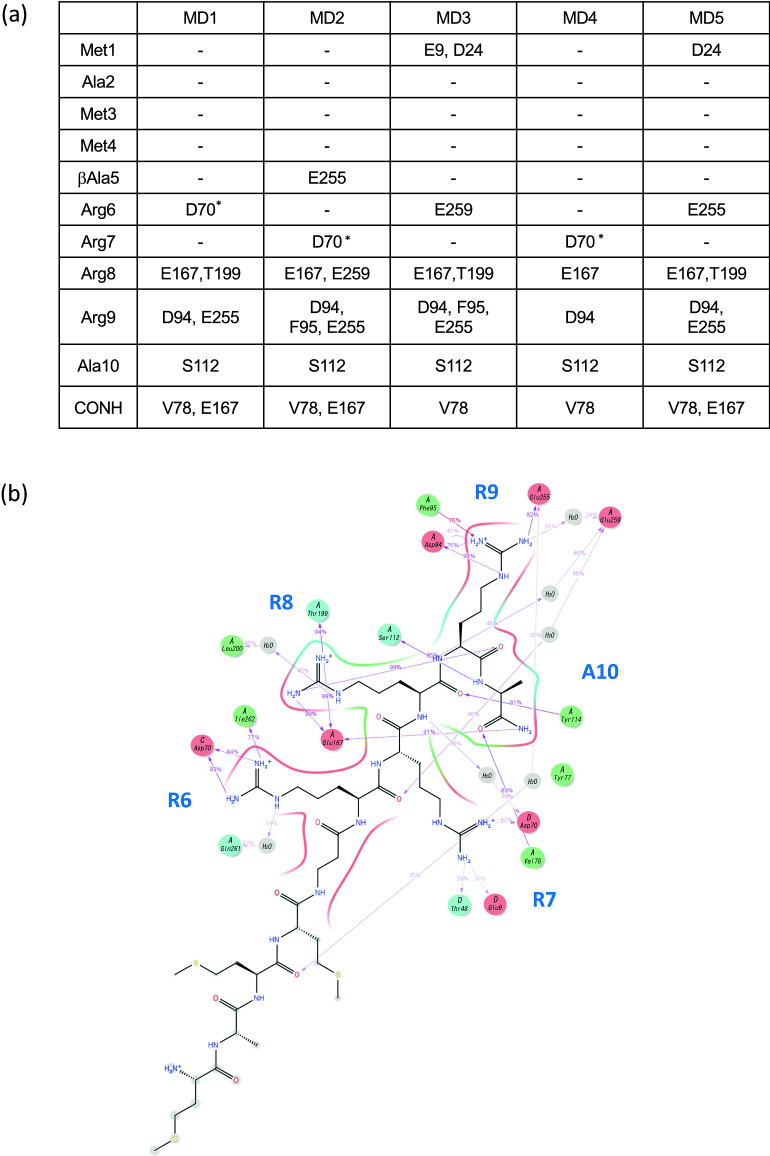

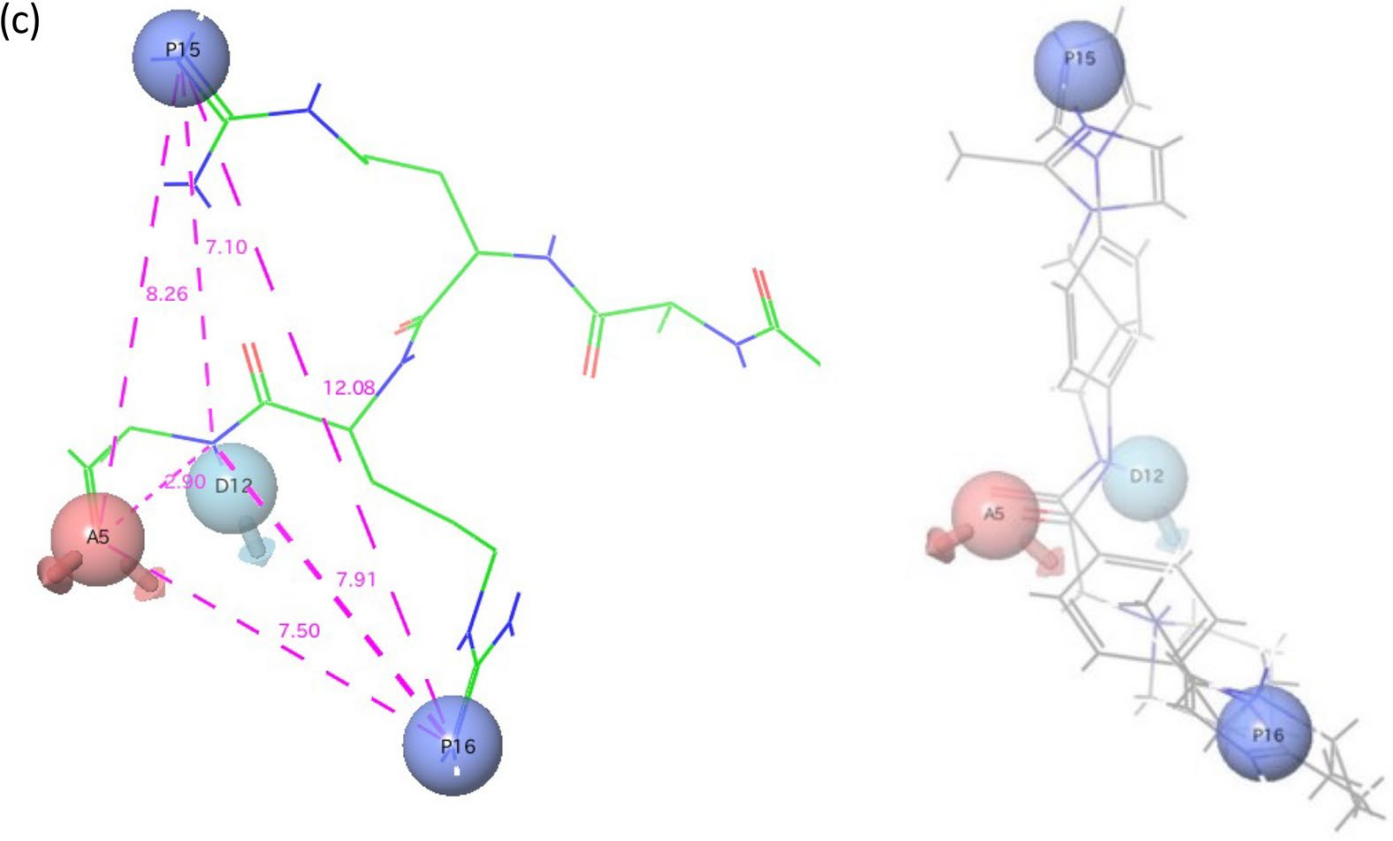
Molecular dynamics (MD) simulations for binding between Stx2a and MMβA-mono. (**a**) Interaction analysis summary from MD simulations of binding between the Stx2a A-subunit and MMβA-mono is shown. Five independent simulation trials (MD1–MD5) were performed. For each MD simulation, Stx2a holotoxin amino acid residues showing more than 80% interaction probability with MMβA-mono are shown. Asterisks indicate residues in the Stx2a B-subunit. (**b**) Scheme showing detailed interactions obtained from MD1. Dotted lines indicate interactions between side chains and the inhibitor, and solid lines indicate interactions between main chains and the inhibitor. Schemes obtained from the other four simulations are shown in Supplementary Fig. S2. The schemes were created using the “Simulation Interactions Diagram” tool in Maestro ver. 2016-2 (https://www.schrodinger.com/products/maestro). (**c**) Pharmacophore model for binding to the A-subunit was determined based on the consensus interaction model of Arg8-Arg9-Ala10 of MMβA-mono and is shown in the left panel. Right panel shows results from pharmacophore screening of a small molecule database. Red sphere, hydrogen bond acceptor; light blue sphere, hydrogen bond donor; deep blue sphere, positive charge. The schemes were created using Maestro ver. 2016-2.

In parallel with our experiments using the shorter MMβA-mono-based peptides, we performed structural analysis of the binding between Stx2a and MMβA-mono using MD simulations. Based on five independent simulation trials (Fig. 3a), we observed substantial interactions between the Arg6-Arg7-Arg8-Arg9-Ala10 region of MMβA-mono and Stx2a. In contrast, interactions between Stx2a and the Met1-Ala2-Met3-Met4-βAla5 region were not detected. In all cases, Arg8 and Arg9 were found to electrostatically interact, respectively, with Glu167 and Asp94 of the A-subunit (Fig. 3b and Supplementary Fig. S2). In addition, simulations show that the main chain of Ala10 tightly interacts with Val78 and Ser112 of the A-subunit via hydrogen bonding. The carboxyl-terminal amide of Ala10 was also found to electrostatically interact with Glu167 of the A-subunit in three trials. Electrostatic interaction between Arg7 and Asp70 of the B-subunit was observed in two trials, whereas interaction between Arg6 and Asp94, which was suggested by X-ray crystallography analysis, was not observed. These results indicate that Arg8-Arg9-Ala10, which corresponds to R2A-mono, is a minimal essential motif for binding to the A-subunit, consistent with results obtained from biochemical and X-ray crystallography analyses. A pharmacophore model was therefore constructed based on conformations of the Arg8-Arg9-Ala10 region of MMβA-mono adopted during MD simulations, using functional groups of MMβA-mono showing an interaction probability of 80% or more with an Stx2a residue in all simulations (Fig. 3c, left panel).

### Pharmacophore screening and docking simulations identify a compound that efficiently inhibits Stx2a in vitro and in vivo

**Figure 4:**
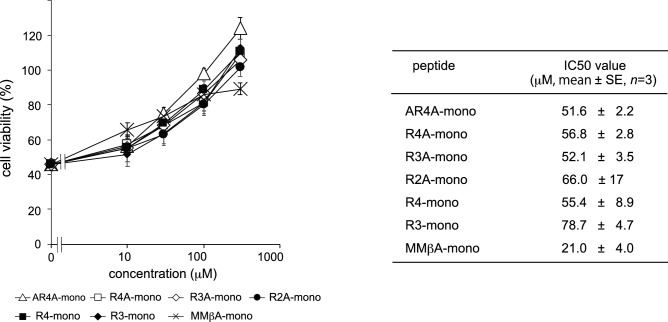
Shorter MMβA-mono-derived peptides efficiently inhibit cytotoxicity of Stx2a. Vero cells were treated with Stx2a for 72 h in the presence of each peptide, and cell viability was measured (left panel). Data are shown as percentage of the control value (mean ± SE, *n* = 3–7). Peptide IC50 values (i.e., the concentrations that restore viability to 50% of the cells killed with no peptide added) are shown at right.

**Figure 5:**
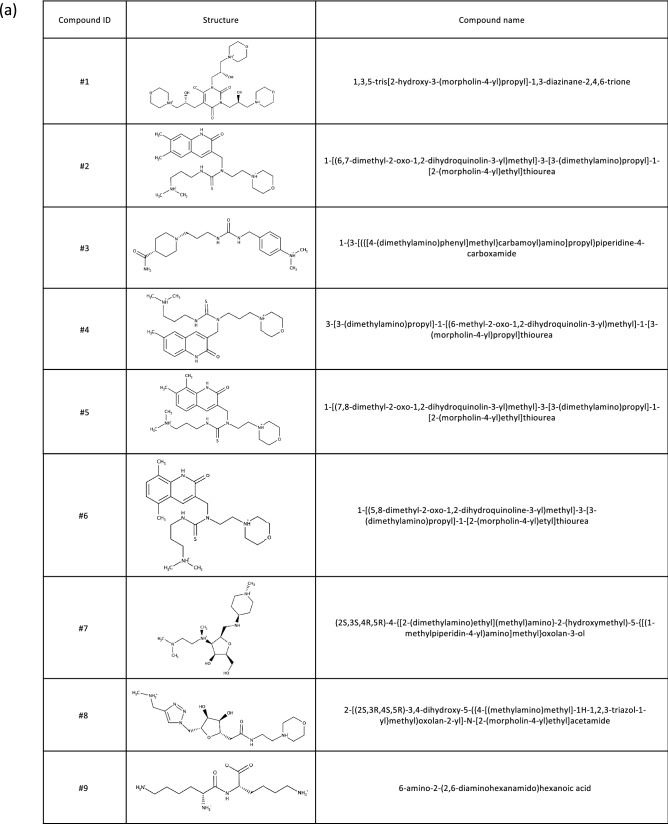

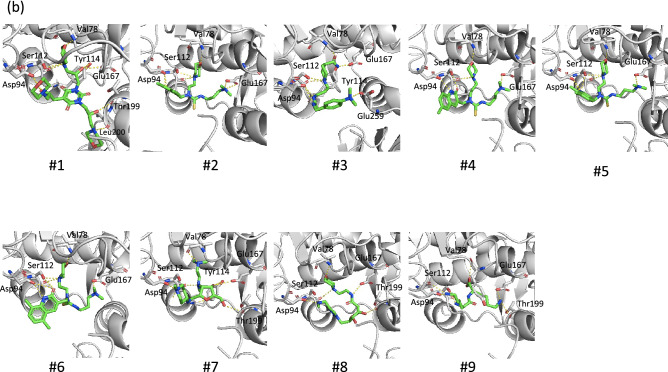
Structures of nine compounds selected by virtual screening. (**a**) The structures and names of compounds #1–#9 identified from docking simulations between molecules identified in pharmacophore virtual screen and the Val78, Asp94, Ser112, and Glu167 residues in the catalytic pocket of the A-subunit. (**b**) Structural view of binding between the Stx2a A-subunit and each compound. Structure images were created using PyMOL ver. 2.3.4, (https://pymol.org/2/).

**Figure 6:**
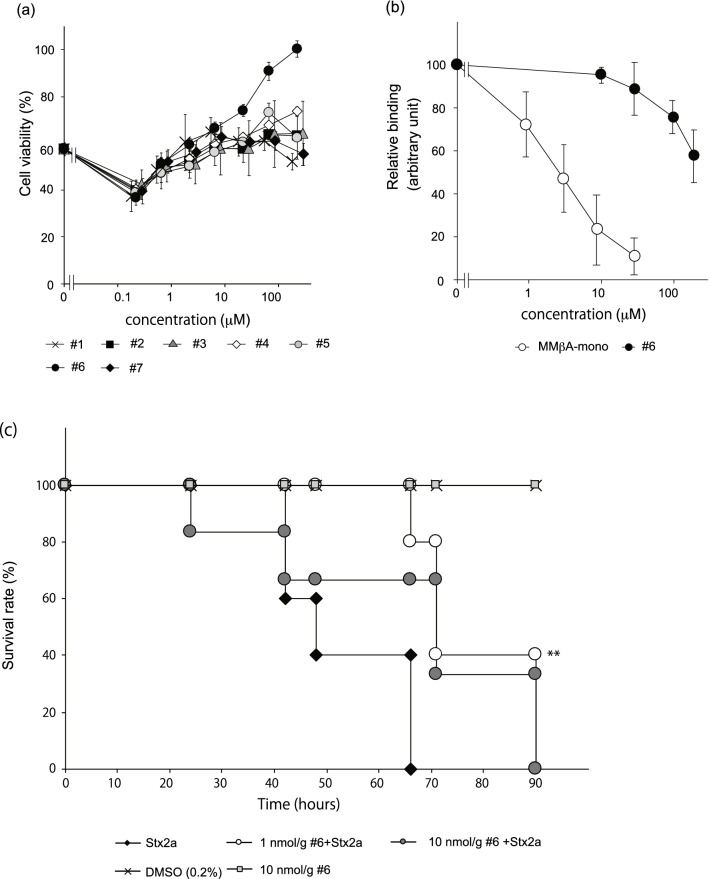
Compound #6 inhibits toxicity of Stx2a. (**a**) Vero cells were treated with Stx2a for 72 h in the presence of each compound. Data are presented as a percentage of the control value (mean ± SE, *n* = 3). (**b**) The AlphaScreen assay was used to measure the inhibitory effects of non-tagged MMβA-mono or compound #6 (dissolved in final 0.4% DMSO) on binding between the Stx2a A-subunit and MMβA-mono. Data are presented as a percentage of the control value without peptides (mean ± SE, *n* = 3). (**c**) Mice were intravenously administered a lethal dose of Stx2a (0.5 ng/g of body weight; *n* = 5) alone or with compound #6 (1 or 10 nmol/g of body weight; *n* = 5 and 6, respectively). Control mice were treated with 0.2% dimethyl sulfoxide (DMSO)–phosphate-buffered saline (PBS) (*n* = 5) or compound #6 (10 nmol /g of body weight; *n* = 5) alone. Data indicate the survival rate of each group during the first 4 days after exposure. *P < 0.05 compared with Stx2a, as determined by Log-rank test.

We next measured the inhibitory effects of the shorter peptides on cytotoxicity of Stx2a. All peptides, including R2A-mono, were found to efficiently inhibit Stx2a toxicity (Fig. 4), confirming that R2A-mono, which is a minimal essential motif for binding, is sufficient for effective inhibition of Stx2a. We then performed virtual screening based on the obtained pharmacophore and identified 768 compounds out of a chemical compound database containing over 7,400,000 molecules (Fig. 3c, right panel). Subsequently, these compounds were subjected to docking simulation to identify those that can interact with Val78, Asp94, Ser112, and Glu167 in the catalytic pocket of the A-subunit. We identified nine molecules, compounds #1–9, that can interact with these four amino acids based on the pharmacophore (Fig. 5a,b). The inhibitory effects of seven commercially available compounds, #1–7, on Stx2a cytotoxicity were then examined. Of these, we found that compound #6 markedly inhibits cytotoxicity, whereas only mild inhibitory effects were observed for the other compounds (Fig. 6a). Compound #6 was also found to inhibit binding of MMβA-mono to the A-subunit (Fig. 6b), indicating that it directly binds to the catalytic cavity of Stx2a. Furthermore, we found that mice intravenously treated with a lethal dose of Stx2a plus compound #6 (1 or 10 nmol/g of body weight) display a longer average survival period than mice treated with Stx2a alone (2.8 days vs. 2.2 days, respectively), indicating that this compound can significantly inhibit lethality of Stx2a (Fig. 6c).

## Discussion

In this study, we identified R2A-mono as the minimal essential motif of MMβA-mono that binds to the A-subunit of Stx2a. Using X-ray crystal structural analysis, we clearly show direct binding between R2A-mono and the A-subunit, in a manner that is identical to that of the Arg8-Arg9-Ala10 region of full-length MMβA-mono^18^. The critical role of Arg8-Arg9 in Stx2a binding was further confirmed by MD simulations with MMβA-mono, which indicated in all trials that Arg8 and Arg9 electrostatically interact with A-subunit residues Glu167 and Asp94, respectively. In contrast, interaction between Arg6 or Arg7 and Stx2a was observed less frequently. These data are also consistent with our previous observation that Ala substitutions of both Arg8 and Arg9 of MMβA-mono completely abolish interaction with the A-subunit, supporting the relative importance of these Arg residues in binding^18^. In addition, the C-terminal Ala10 of both R2A-mono and MMβA-mono was found to interact with A-subunit residues Val78, Ser112, and Arg170 by X-ray crystal structural analysis and with Val78, Ser112, and Glu167 by MD simulations. Notably, Glu167 and Arg170 are located in close proximity to one another in the binding pocket, and both residues are known to be essential for catalytic activity^23,24^, consistent with the important role of Ala10 in binding.

On the other hand, R4-mono and R3-mono, both of which lack the C-terminal Ala, inhibited the binding of biotinylated MMbA-mono to the A-subunit with more efficacy compared to R4A-mono and R3A-mono, respectively (Fig. 1). Although the precise mechanism of the detrimental effect of the C-terminal Ala is remained to be elucidated, the presence of the amide group of the C-terminal Ala may contribute to fix the binding manner of the peptides with this Ala, such as MMbA-mono, AR4A-mono, R4A-mono, R3A-mono, and R2A-mono, to the same orientations which are clearly demonstrated in their crystal structures (Fig. 2). In contrast, the binding manner of R4-mono and R3-mono may be more flexible but strong enough to compete the binding with more efficacy, consistent with our observation that electron densities for R3-mono and R4-mono were not determined in X-ray crystallography analysis performed with co-crystals of these peptides and Stx2a holotoxin.

We further constructed a pharmacophore model based on the conformations adopted by Arg8-Arg9-Ala10 during our MD simulations, in which the pharmacophores are functional groups of MMβA-mono showing high probability for interaction with Val78, Asp94, Ser112, and Glu167 of the A-subunit in all simulations. Previously developed small compounds targeting Stx, such as purine- or amide-derivatives^19,25^, were obtained by high-throughput screening of chemical libraries via targeting of the “adenine-specificity” pocket, and therefore, they mainly interact with Val78, Ser112, Tyr114, and Arg170^19^. However, none of these compounds has been shown to interact with Asp94, which is present in the gate area of the pocket. In contrast, the clear interactions we observe between Arg8 and Glu167 and between Arg9 and Asp94 yielded unique pharmacophores covering a wide region of the catalytic pocket from the bottom of the cavity (Glu167) to the gate area (Asp94). Compared to previously developed compounds, this feature enabled identification of a series of relatively large molecules, which inhibit the toxin with more specificity and efficacy. Notably, docking simulations with the 768 compounds identified by screening with our pharmacophore model yielded nine compounds predicted to interact with Val78, Asp94, Ser112, and Glu167. Of these, we show that compound #6 interacts with Stx2a in the same manner as MMβA-mono, which may contribute to the strong inhibitory effect observed for this molecule in vitro and in vivo.

Consistently, we further find that R2A-mono, as well as the other MMβA-mono-derived peptides, efficiently inhibit Stx2a cytotoxicity. This confirms that the Arg8-Arg9-Ala10 motif is sufficient for effective inhibition of Stx2a, although in competition assays, the relative IC50 value of R2A-mono was found to be much higher than that of the other peptides (Fig. 1). Although the precise inhibitory mechanism remains to be elucidated, the small size and basic nature of R2A-mono may lead to high cell-permeability, as in general, clustered Arg residues can penetrate into cells^26^. This would allow targeting of the A-subunit, which is present in cells after B-subunit-mediated endocytosis of the toxin. Similarly, the hydrophobicity of compound #6 may facilitate penetration through the cell envelope and contribute to highly efficient toxin inhibition.

In summary, here we determined the minimal binding motif for the monomeric peptide MMβA-mono to Stx2a. In addition, the pharmacophore modeled from our observed interactions between Stx2a A-subunit and both the R2A-mono peptide and the R2A region of MMβA-mono successfully identified a compound (#6) that shows promise as a possible therapeutic agent against EHEC infection. We further propose that this pharmacophore may also be applicable to the design of highly selective inhibitors against the potent bioterrorism agent ricin, an RIP that can be isolated from the seeds of the castor plant, *Ricinus communis*, and whose catalytic region adopts a structure highly similar to that of Stx^27^.

## Methods

### Preparation of recombinant Stx2a and dissociated A-subunit

Recombinant Stx2a was prepared as described previously^28^. The Stx2a A-subunit was prepared as follows: purified Stx2a was incubated in dissociation solution containing 6 M urea, 0.1 M NaCl, and 0.1 M propionic acid (pH 4), and each dissociated subunit was separated by gel filtration column chromatography (Sephacryl S-200; Cytiva, Marlborough, MA, USA). Fractions containing the A-subunit were dialyzed against 50 mM Tris–HCl (pH 7.4).

### Peptides

Peptides were synthesized as described previously^16,18^. In brief, monomer peptides were synthesized from *N*-α-Fmoc-protected amino acids with standard BOP/HOB coupling chemistry, using TentaGel amide resin (Intavis Bioanalytical Instruments AG, Cologne, Germany). The C-terminus of the obtained peptide is amidated. To biotinylate peptides, terminal amino groups were treated with biotin (Sigma-Aldrich, St. Louis, MO, USA) and 1-(bis[dimethylamino]methylene)-1*H*-benzotriazolium 3-oxide hexafluorophosphate (Peptide Institute Inc., Osaka, Japan) in the last cycle of peptide synthesis. Synthesized peptides were validated by mass spectrometry analysis using the AutoflexII TOF/TOF system (Bruker Corp., Billerica, MA, USA). Peptide concentration was determined based on the weight of the lyophilized peptide powder.

### Competition assays with shorter MMβA-mono-derived peptides to determine their effect on binding between Stx2a A-subunit and MMβA-mono

The inhibitory effects of a series of shorter MMβA-mono-derived peptides on binding between the Stx2a A-subunit and MMβA-mono were measured using the AlphaScreen assay, as described previously^18^. Briefly, biotinylated MMβA-mono (30 nM) was incubated with Stx2a A-subunit (20 nM) in the presence of indicated concentrations of a single shorter peptide and specific anti-Stx2a A-subunit monoclonal antibody (originally obtained) in individual wells of an OptiPlate-384 (PerkinElmer, Waltham, MA, USA) for 30 min at room temperature. Samples were then incubated with anti-IgG (protein A) acceptor beads (20 µg/ml; PerkinElmer) for 30 min, followed by incubation with streptavidin donor beads (20 µg/ml; PerkinElmer) for 1 h at room temperature in the dark. The plate was then subjected to excitation at 680 nm, and emission from wells was monitored at 615 nm with the EnVision system (Perkin Elmer). Data were obtained as arbitrary units (AUs) of signal intensity (counts per second). IC50 values were determined by using Image J software ver. 1.53 k.

### Crystallization

Crystallization of Stx2a was performed as described previously^18^. In brief, purified Stx2a holotoxin was concentrated to 4–8 mg/ml in 0.2 M NaCl and 25 mM potassium phosphate (pH 6.5), using Amicon Ultra-0.5 Centrifugal Filters (10 kDa cutoff). Crystallization conditions were as follows: 4.0 M sodium formate, 100 mM 2-(N-morpholino) ethanesulfonic acid (MES) (pH 6.5), 50 mM 3-(1-Pyridinio)-1-propanesulfonate (PPS), and 2% ethylene glycol. The micro-seeding method was used to obtain crystals with high reproducibility. To prepare complexes with a series of shorter MMβA-mono-derived peptides, Stx2a holotoxin crystals were soaked with 5 mM of each peptide in artificial mother liquor, containing 4.0 M sodium formate, 70 mM MES (pH 6.5), 35 mM PPS, and 1.4% (v/v) ethylene glycol, for 1.5 h. The crystals were then cryoprotected in cryoprotectant solution [30% (v/v) glycerol, 2.8 M sodium formate, 70 mM MES pH 6.5, 35 mM PPS] containing 5 mM of each peptide for 15 s.

### Diffraction data collection and structure determination

Diffraction data for peptide complex crystals were collected at 95 K on beamlines BL-17A and BL-17A of the Photon Factory (PF) at the High Energy Accelerator Research Organization (KEK; Tsukuba, Japan). Diffraction data were processed and scaled using the programs *XDS* and *aimless*^29,30^. All crystals complexed with AR4A-mono, R4A-mono, R3A-mono, and R2A-mono belonged to space group *P*6_1_ (Table 1). The crystal structures of the complex with AR4A-mono (PDB ID: 7VHC), R4A-mono (PDB ID: 7VHD), R3A-mono (PDB ID: 7VHE), and R2A-mono (PDB ID: 7VHF) were determined by the molecular replacement (MR) method, using the PHENIX program 1.19_4092^31^. The PDB coordinates of 1R4P (Shiga toxin type 2)^15^ were used as a search model for the MR calculations. Crystallographic refinements were performed using the phenix.refine program^31^, and the surface electrostatic potential was calculated in PyMOL ver. 2.3.4 (Schrödinger, Inc., New York, NY, USA).

### MD simulations for pharmacophore modeling and virtual screening

To prepare Stx2a and MMβA-mono complex structure for MD simulation, assignment of bond orders and hydrogenation were performed using Maestro (Schrödinger Release 2016–2, Schrödinger, Inc.). Disordered regions and side chains of Stx2a and the structure of MMβA-mono were repaired using Prime^32^. The suitable ionization states of each ligand were generated by Epik^33^ at pH 7.0 ± 2.0. Hydrogen bond optimization was performed using PROPKA^34^, and energy minimization calculations were conducted with Maestro, using the OPLS2005 force field^35^.

Set-up for MD simulations was performed by the Molecular Dynamics System Setup Module in Maestro. The prepared Stx2a and MMβA-mono complex structure was placed in an orthorhombic box with a buffer distance of 10 Å to create a hydration model, and the SPC water model^36^ was used for constructing the hydration model. NaCl (0.15 M) served as the counter ion to neutralize the system. MD simulations were performed by Desmond^37^, with the cutoff radii for van der Waals interactions set to 9 Å, and the time step, initial temperature, and pressure of the system set to 2.0 femtoseconds, 300 K, and 1.01325 bar, respectively. The sampling interval during the simulation was set to 1 picosecond, and simulations were performed using the NPT ensemble for 50 ns. Using the constructed hydration model, five simulations were performed at different initial velocities. All trajectories from MD simulations were aligned to the initial structure with protein Cα, and the Simulation Interactions Diagram tool in Maestro was used to perform an interaction analysis between Stx2a and MMβA-mono.

Functional groups of MMβA-mono that showed an interaction probability of 80% or more with an amino acid residue of Stx2a in all simulations were defined as pharmacophores. Approximately 7.4 million compounds from the Namiki Shoji Co., Ltd. (Tokyo, Japan) database were used for pharmacophore screening with Phase^38^ software. After screening, all pharmacophore-matched compounds were reevaluated by docking simulation with Stx2a. For this analysis, a grid box with dimensions of 10 × 10 × 10 Å^3^ was generated with the MMβA-mono centroid specified, and docking simulation was performed with Glide in standard-precision mode^39,40^. We then selected compounds with a docking pose showing interactions with Val78, Asp94, Ser112, and Glu167. All applications were used as provided in Maestro ver. 2016-2 (Schrödinger, Inc).

### Cytotoxicity assay

Cytotoxicity assay was performed as described previously^18^. In brief, subconfluent Vero cells were cultured in 96-well plates in Dulbecco’s modified Eagle medium, supplemented with 10% fetal calf serum, 100 units/ml penicillin, 100 μg/ml streptomycin, and 0.25 μg/ml amphotericin B. Cells were then treated with Stx2a (3 pg/ml) in the absence or presence of a given peptide or compound for 72 h at 37 °C. The relative number of living cells was determined using a Cell Count Reagent SF (Nacalai Tesque, Kyoto, Japan), according to manufacturer instructions.

### Toxicity of Stx2a in mice

A lethal dose of Stx2a (0.5 ng/g of body weight) was intravenously administered to female ICR mice (18–20 g, Japan SLC, Japan) with or without the indicated amount of compound #6 dissolved in 0.2% dimethyl sulfoxide (DMSO)–phosphate-buffered saline (PBS). Control mice were treated with compound #6 or 0.2% DMSO–PBS alone. The survival periods of all mice were monitored, and the data were subjected to Kaplan–Meier survival analysis. All animal experiments were approved by the Animal Ethics Committee of Doshisha University prior to their commencement and were performed in accordance with approved protocols. All animal experiments were performed in accordance with the ARRIVE guidelines.

### Statistics and reproducibility

Significant differences of survival rate were analyzed using the Log-rank test. Statistical analysis was performed using IBM SPSS Statistics software (ver. 27.0.0.0). No statistical methods were used to determine sample size. Each experiment was performed at least three times to confirm the reproducibility of our results.

## Supplementary Information

Supplementary Figure 1.

Supplementary Figure 2.

## Data Availability

All source data presented in the main figures and supplementary figures are available in Supplementary Data 1. The structure datasets generated and/or analyzed in the current study are available in the PDB repository under accession numbers 7VHC, 7VHD, 7VHE and 7VHF. All other data or sources are available from the corresponding authors on reasonable request.
