# Supplementary material for: A unique peptide-based pharmacophore identifies an inhibitory compound against the A-subunit of Shiga toxin
Source: Sci Rep. 2022 Jul 6;12:11443. doi: 10.1038/s41598-022-15316-1 (PMC9259562; doi:10.1038/s41598-022-15316-1)
Supplement: Supplementary file 1 — Supplementary Figure 1. [file 41598_2022_15316_MOESM1_ESM.pdf]

Supplementary Information

A unique peptide-based pharmacophore identifies an inhibitory compound against the A-subunit of Shiga toxin

Miho Watanabe-Takahashi[1,#], Miki Senda[2,#], Ryunosuke Yoshino[3, 4#], Masahiro Hibino[1], Shinichiro Hama[1], Tohru Terada[3], Kentaro Shimizu[3,*], Toshiya Senda[2, 5*], and Kiyotaka Nishikawa[1,*]

[1]Department of Molecular Life Sciences, Graduate School of Life and Medical Sciences, Doshisha University, Kyoto, Japan.
[2]Structural Biology Research Center, Institute of Materials Structure Science, High Energy Accelerator Research Organization (KEK), Ibaraki, Japan.
[3]Department of Biotechnology, Graduate School of Agricultural and Life Sciences, The University of Tokyo, Tokyo, Japan
[4] Transborder Medical Research Center, University of Tsukuba, Ibaraki, Japan
[5]Department of Materials Structure Science, School of High Energy Accelerator Science, The Graduate University of Advanced Studies (Soken-dai), Ibaraki, Japan.

#These authors contributed equally.
*Correspondence should be addressed to K.S. (for molecular dynamics and simulations; shimizu@bi.a.u-tokyo.ac.jp), T.S. (for crystallographic study; toshiya.senda@kek.jp), and K.N. (knishika@mail.doshisha.ac.jp)

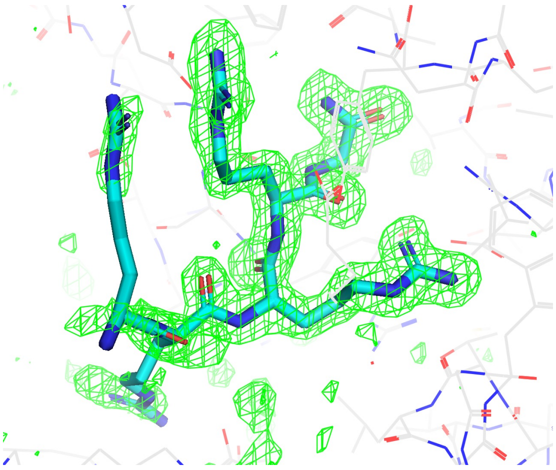

AR4A-mono

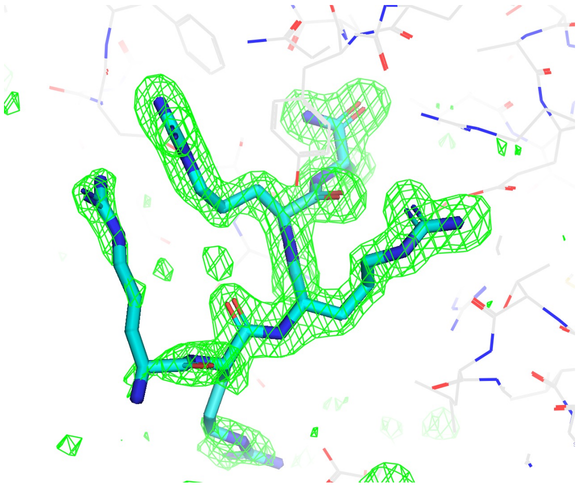

R4A-mono

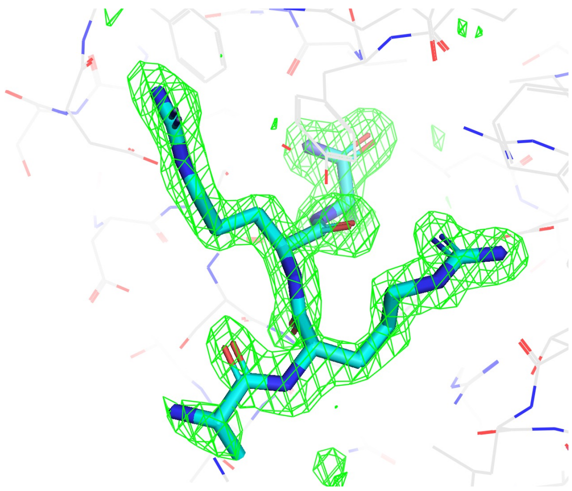

R3A-mono

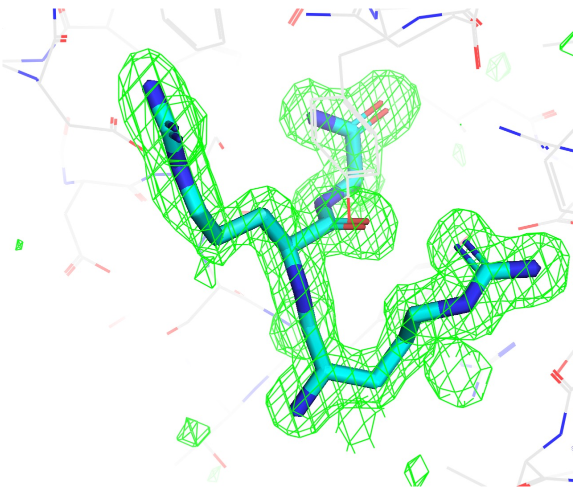

R2A-mono

Supplementary Fig.1
Simulated annealing mFo-DFc omit map for bound peptide in Stx2a. The positive mFo-DFc electron density of the peptide was contoured at 3 sigma. AR4A-mono, R4A-mono, R3A-mono, and R2A-mono are shown in light blue.
