# Supplementary material for: A unique peptide-based pharmacophore identifies an inhibitory compound against the A-subunit of Shiga toxin
Source: Sci Rep. 2022 Jul 6;12:11443. doi: 10.1038/s41598-022-15316-1 (PMC9259562; doi:10.1038/s41598-022-15316-1)
Supplement: Supplementary file 2 — Supplementary Figure 2. [file 41598_2022_15316_MOESM2_ESM.pdf]

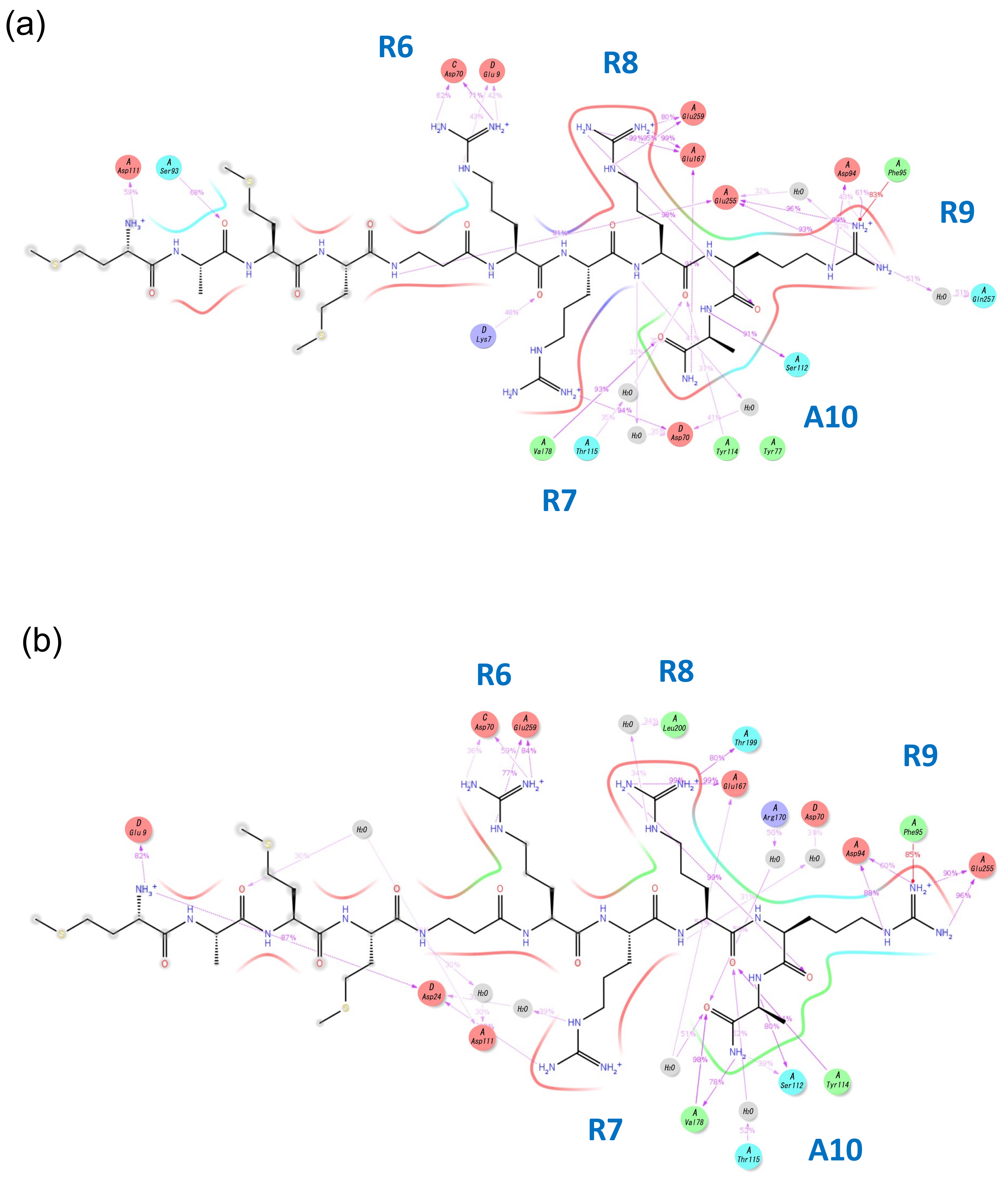

Supplementary Fig.2

(c)

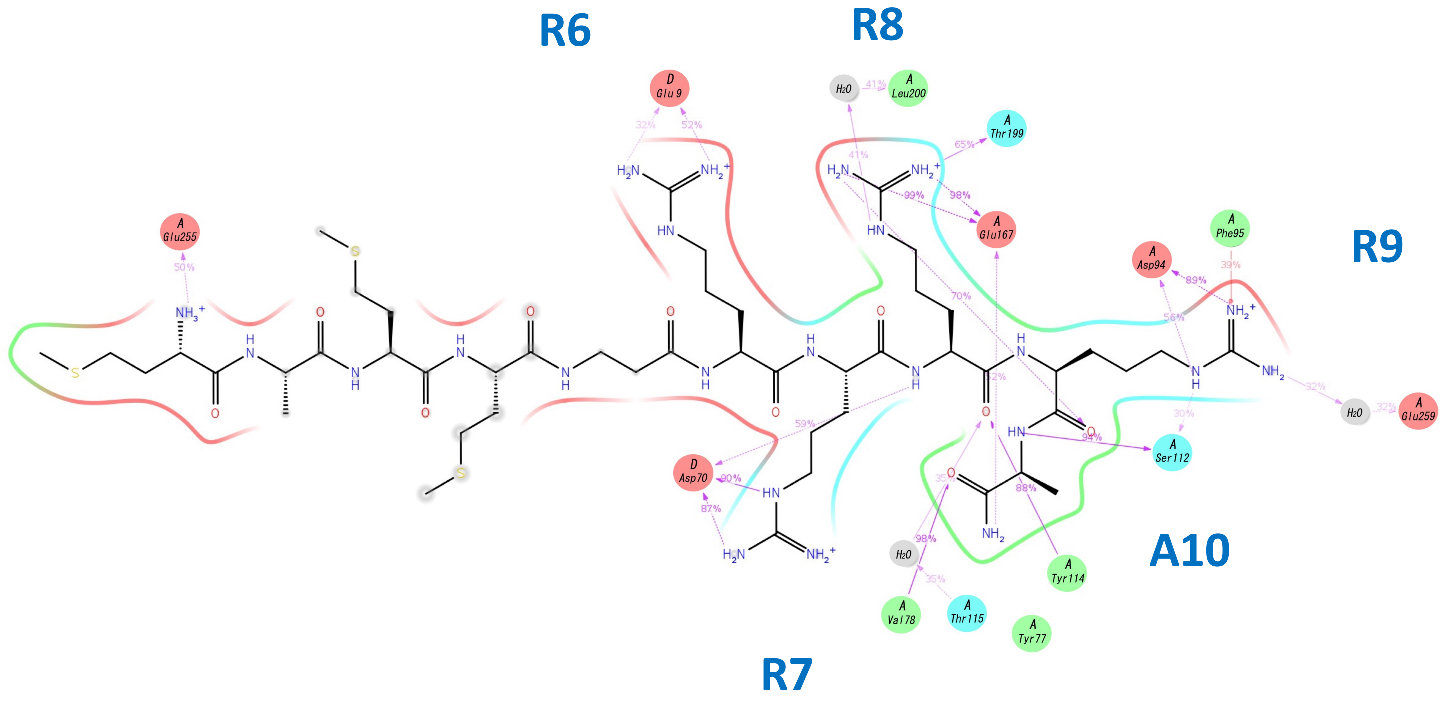

(d)

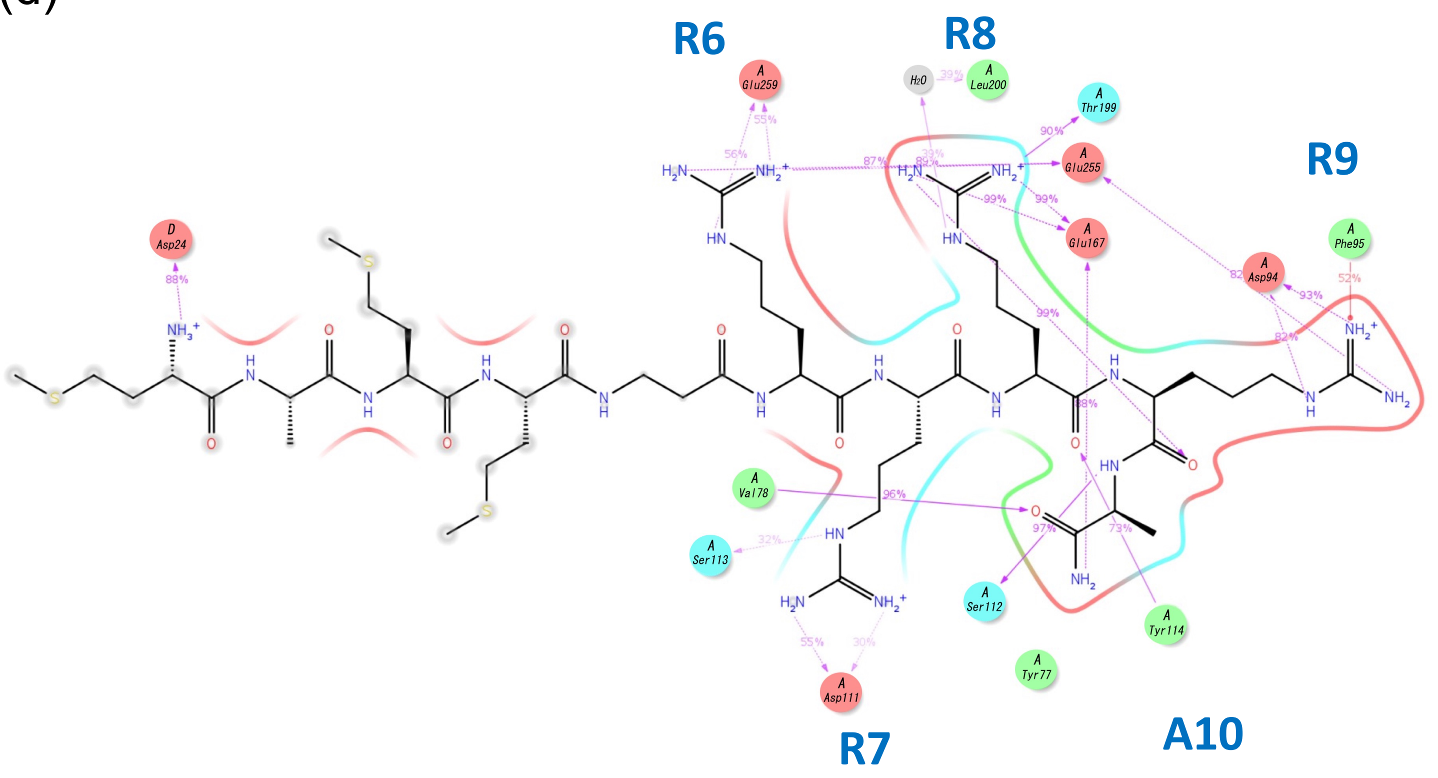

Supplementary Fig.2
Scheme of detailed interactions obtained from (a) MD2, (b) MD3, (c) MD4 and (d) MD5 are shown.
